# An immunohistochemical examination of cinnamon extract administration on distribution of NGF (nerve growth factor) and Trk-A (tyrosine kinase-A) receptor for diabetic rats with pancreatic tissue

**DOI:** 10.3906/sag-2012-270

**Published:** 2021-10-21

**Authors:** Şükran YEDİEL ARAS, Ebru KARADAĞ SARI

**Affiliations:** 1 Department of Midwifery, Faculty of Health Sciences, Kafkas University, Kars Turkey; 2 Department of Histology-Embryology, Faculty of Veterinary Medicine, Kafkas University, Kars Turkey

**Keywords:** Cinnamon, diabetes, female and male rats, NGF, pancreas, Trk-A

## Abstract

**Background/aim:**

The aim of this study was to investigate the administration of cinnamon extract that is known to be effective in decreasing the high blood glucose and the distribution of NGF and Trk-A receptor in pancreas with immunohistochemistry way.

**Materials and methods:**

The experimental groups were defined as control, sham, cinnamon, diabetes, and diabetes-cinnamon. At the end of the experiment, the pancreatic tissue samples were obtained for the rats. The hematoxylin-eosin and triple staining were used to examine histology. The immunohistochemical methods were performed on the sections of pancreatic tissue. In all groups, the body weight and fasting blood glucose obtained from the male and female rats and the values were statistically evaluated.

**Results:**

The NGF immunoreactivity was observed in acinus, excretory pars, excretorius ducts, and islets of Langerhans for the pancreatic tissues of female and male rats in all groups. The Trk-A immunoreactivity was observed in acinus and islets of Langerhans for the pancreatic tissues of female and male rats in the control, sham, and cinnamon groups.

**Conclusion:**

As a result, it was determined that the cinnamon, which is effective on blood glucose levels, has a positive effect on the NGF production in pancreas.

## 1. Introduction

In diabetes which is a chronic metabolic disease, lack of insulin or problems in insulin utilization occur [1]. It was stated that the significant reduction in the beta (β) cell found in the pancreas has an important role in the appearance of diabetes. The diabetes disease has two types. While the immune destruction of all β cells occurs in type-1 diabetes, the β cells are partially destructed in type-2 diabetes [2]. The cinnamon is a native herb belonging to Sri Lanka and is also a tropical Asian spice derived from the inner bark of several trees of the Cinnamomum genus. Some types of the cinnamon are used not only as a spice in foods, but also in traditional and modern medicine. Both Ceylon cinnamon (cinnamon oil) and Chineese cinnamon oils are used in the pharmaceutical production [3–5]. The cinnamon was reported that it reduces the high blood glucose level, repairs the damaged β cells, and has positive effects on the diabetes [6,7]. The neurotrophins (NTs) are the growth factors with polypeptide structure. The neurotrophins are necessitated for controlling of the synaptic functions and plasticity and maintaining of the neural life morphology and differentiation. However, the neurotrophins were remarked to have different functions in the systems apart from the nerve system [8,9]. The nerve growth factor (NGF) is one of the first discovered members of the neurotrophin family. The NGF was defined as a trophic protein that has a role in different processes such as neuroblast proliferation, dorsal root ganglion maturation and axonal growth and acts as a message-receiver between the tissues that react against the peripheral stimulation and nerves stimulating these tissues [9–12]. The NGF and Trk-A receptors have important roles in the development, differentiation, survival and regulation of neurotransmitters of the peripheral and central nerves in the embryonic and postnatal life [13]. The NGF was reported to attach to two types of receptors located on the cell surface. One of these receptors is known as tyrosine kinase A (Trk-A) and the other one is known as p75, which does not have a tyrosine kinase activity [14,15]. The NGF synthesis does not only arise from the target tissues of central and peripheral nerve system, but also from the mast cells, lymphocytes, lipid tissue cells, pancreatic β cells, hair follicles, thymus, spleen [16–20], smooth muscle cells, fibroblasts [10,11], and trigeminal mesencephalic neurons [21]. The aim of this study is to determine the NGF and Trk-A levels in pancreatic tissue on cinnamon extract administration to diabetic rats by immunohistochemical methods.

## 2. Materials and methods

### 2.1. Animals

The approval for the research was received from Cumhuriyet University Animal Experimentation Local Ethics Committee with 65202830/118 board number on 19.06.2014. The animals used in the research were supplied from Ataturk University Laboratory Animals Unit. 

### 2.2. Method

We designed a prospective experimental study. In this research, 60 (30 males + 30 females) one-month old Sprague dawley rats were used. The rats were housed at 25 ± 2 °C room temperature and 60%–65% humidity conditions for 12 h in dark cycle and for 12 h in light cycle in the cages that are cleaned daily. This study complies with International Helsinki Declaration principles. All rats planned to be used in the experiment were weighed. Rats were randomly divided into five groups. The groups were formed as described below:

3. Control Group (n = 12) (6 females+6 males): The rats were fed ad-libitum with normal rat food.

4. Sham Group (n = 12) (6 females+6 males): 50 mg/kg of sodium citrate solution were intraperitoneally applied (i.p.).

5. Cinnamon Group (n = 12) (6 females+6 males): The cinnamon extract was given to the cinnamon groups as oral gavage method for 14 days (200 mg/kg) by dissolving in 1 mL of distilled water [22].

6. Diabetes Group (n = 12) (6 females + 6 males): The streptozotocin (STZ) (the streptozotocin was dissolved in the citric acid and disodium hydrogen phosphate buffer and pH was 4.5) was intraperitoneally applied for only one dose as 50 mg/kg. The animals were considered as diabetic, if their blood glucose values were above 250 mg/dL on the third day after the STZ injection [23].

7. Diabetes+Cinnamon Group (n = 12) (6 females+6 males): The streptozotocin (STZ) was dissolved in the citric acid and disodium hydrogen phosphate buffer while the pH was 4.5) was intraperitoneally applied for only one dose as 50 mg/kg. The animals were considered as diabetic, if their blood glucose values were above 250 mg/dL on the third day after the STZ injection. The cinnamon extract was applied by oral gavage method for 14 days (200 mg/kg) [22,23]. 

At the end of the study, the body weights and blood glucose of the rats were measured and then, they were taken to the deep sedation with disinfection by using an anesthetic substance named Sevoflurane, and the pancreatic tissue samples were subsequently obtained. At the end of the study, the blood glucose levels were measured by taking from the tail vein of the rats fasted for 8 h.

#### 2. 2. 1. Preparation of cinnamon extract

Ceylon cinnamon was used in the study in powder form. Ten grams of powdered cinnamon were taken and dissolved in 100 mL of ethanol. The mixture obtained was left to shake on a shaker for 24 h at the room temperature. After the time expired, the mixture was filtered by the help of a filter paper. The solvent content was removed in the rotary evaporator at the low pressure and low temperature. The extracts were stored at –20 °C until the experiment [24]. The cinnamon extract was applied by dissolving in 1 mL of distilled water.

#### 2. 2. 2. Histological examinations

The pancreatic tissue samples were fixed within 10% formalin solution. Following the routine procedures, they were embedded into the paraffin and 5 μm sections were obtained from the paraffin blocks. In order to demonstrate the histological structure of pancreatic tissue, the sections were performed by Crossman’s triple staining and hematoxylin-eosin (HE) staining [25] methods and examined under a light microscope (Olympus BX51; Olympus Optical Co. Osaka, Japan).

#### 2. 2. 3. Immunohistochemical examinations

The sections obtained from the paraffin blocks were incubated in 3% H_2_O_2_ (hydrogen peroxide) and prepared in 0.1 M of phosphate buffered saline (PBS) for 15 min to inhibit the endogenous peroxidase activity after the deparaffinization and rehydration procedures. Then, the sections were washed in the PBS solution. The NGF was applied to the tissues in order to release the antigenic receptors that were boiled by a microwave for 10 min at 600 watts in a citrate buffer solution (pH: 6.0), the Trk-A was applied to the tissues in order to release the antigenic receptors that were boiled by a microwave for 10 min at 600 watts in a tris-EDTA Buffer solution (pH: 6.0). The avidine-biotin-peroxidase protocol was used to investigate the NGF and Trk-A immunoreactivity [26]. The tissues were incubated by Blocking Solution A (Invitrogen-Histostain plus Bulk Kit) for 10 min and then, the NGF (Abcam-AB6198) (1/600 dilution) primary antibody and Trk-A (Abcam-AB76291) (1/400 dilution) primary antibody were diluted by the PBS applied. The tissues were treated by the NGF incubated overnight at +4 °C. The tissues were treated by the Trk-A incubated by the primary antibody at the room temperature for 1 h. Then, the biotinylated secondary antibody and streptavidin-peroxidase solutions (Invitrogen-Histostain plus Bulk Kit) were applied for 30 min. The DAB-H2O2 (diaminobenzidinehidrogenperoxide) [27] was applied to the tissues washed by the PBS as an encolouring substrate. The hematoxylin was applied on the slides for counterstaining after washing with the distilled water. The slides were then dehydrated and covered by the immunmount. The density of immunoreactivity was evaluated semi-quantitatively depending on the severity of reaction and density in the tissue. The evaluation was made by two independent observers. Depending on the staining properties, the slides were scored within the range of 0–3 during their evaluation: weak staining (1), intermediate staining (2), and intense staining (3) [28,29]. All procedures were performed in the same way without adding the primary antibody to the slides in the negative control group. The slides prepared for the histological and immunohistochemical investigations were evaluated and photographed by a light microscope (Olympus BX51, Japan). The number of NGF and Trk-A immunoreactivity positive cells was counted by using the image-j (vI.50i) software. The numerical distribution of NGF and Trk-A positive cells was observed in six different sections chosen from eight unit areas of the islets of Langerhans and acinar cells for each animal [30]. 

#### 2. 2. 4. Statistical analysis

The SPSS (v.20.0) package software was used to evaluate the data obtained in the study. Kruskal–Wallis H test was performed to determine the differences between the groups (control, sham, cinnamon, diabetes, diabetes + cinnamon) for the evaluation of live weight and blood glucose data. Mann–Whitney U test was used to compare the differences between the significant groups. Wilcoxon test was used to determine the differences between the live weights of the rats in the groups on the 1st day and the 17th day. In order to determine the differences between the blood glucose levels of the rats on the 1st, 3rd, and 17th days, the Friedman test was performed first and then the Wilcoxon test between the measurements. The Chi-square test was used to determine the difference between the groups on account of the NGF and Trk-A immunoreactivity positive cells. Results are expressed as median (min–max). P values < 0.05 were considered statistically significant. 

## 3. Results

### 3. 1. Live weight results 

**Figure 1:**
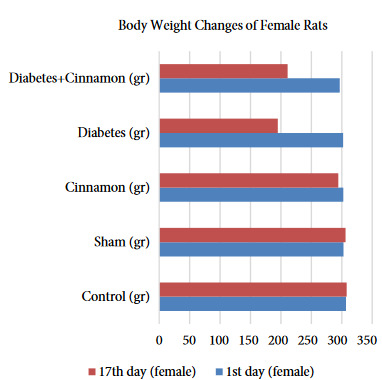
The body weight changes of female rats.

**Figure 2:**
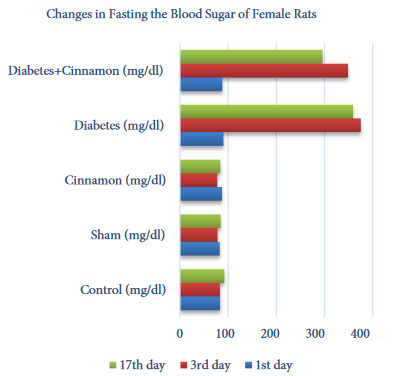
The body weight changes of male rats.

The live weight of male and female rats was statistically evaluated within and between the groups and the results were given as tables and figures. The female rats in the diabetes and diabetes+cinnamon groups did not show any statistically significantly difference on the 17th day of the research in compared to the live weight averages (p > 0.001). However, at the end of the research, the weight averages of diabetes and diabetes+cinnamon groups showed a statistically significant decrease compared to the first-day results (p < 0.001) (Table 1, Figure 1). The male rats in the diabetes and diabetes+cinnamon groups showed a statistically significant difference on the 17th day of the research in compared to the live weight averages (p < 0.001). The weight averages of both groups showed a statistically significant decrease on the 17th day in compared to the 1st day. It was observed that the live weight of rat decreased significantly in the diabetes+cinnamon group (Table 2, Figure 2).

**Table 1:** The statistical evaluation of live weights of the female rats according to the groups.

Days	Control (g)	Sham (g)	Cinnamon (g)	Diabetes (g)	Diabetes+Cinnamon (g)
1st day	305.5 (304–315) aA	297.5 (289–324)aA	296.5 (285–338)aA	312.0 (276–317)aA	297.5 (289–302)aA
17th day	305.0 (296–339) aA	303.5 (296–322)aA	291.0 (277–324)aB	198.5 (151–236)bB	210 (200–226)bB

A, B: As a result of Wilcoxon test; the differences between the averages in the same column expressed with the different letters are statistically significant (p < 0.001), a, b: As a result of the Kruskal–Wallis H test and the Mann–Whitney U test performed afterwards; the differences between the averages in the same line expressed with the different letters are statistically significant (p < 0.001).

**Table 2:** The statistical evaluation of live weights of the male rats according to the groups.

Days	Control (g)	Sham (g)	Cinnamon (g)	Diabetes (g)	Diabetes+Cinnamon (g)
1st day	421 (414–449)Aa	425 (413–436)Aa	429 (424–436)Aa	436 (428–443)Aa	420 (400–437) Aa
17th day	441 (422–450)Aa	439 (425–450)Ba	420 (406–437)Ab	404 (324–325)Bb	307.5 (261–354)Bc

A, B: As a result of Wilcoxon test; the differences between the averages in the same column expressed with the different letters are statistically significant (p < 0.05), a, b, c: As a result of the Kruskal–Wallis H test and the Mann–Whitney U test performed afterwards; the differences between the averages in the same line expressed with the different letters are statistically significant (p < 0.05).

#### 3. 2. Blood glucose levels

**Figure 3:**
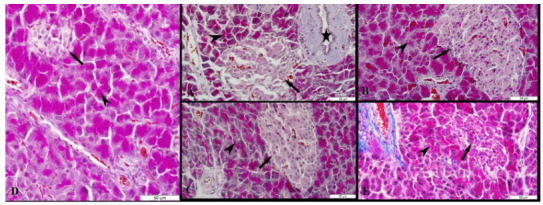
The changes in fasting the blood sugar of female rats.

**Figure 4:**
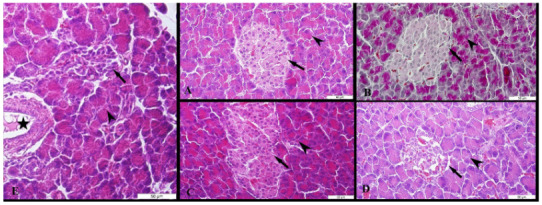
The changes in fasting the blood sugar of male rats.

The blood glucose levels of male and female rats were statistically evaluated within and between the groups and the results were given as tables and figures. The female rats in the diabetes and diabetes+cinnamon groups did not show any statistically significant difference between the 3rd and 17th days in blood glucose levels. But, it was observed that the 17th day average blood glucose levels in the diabetes+cinnamon group decreased though they were not directly reflected by the statistics (Table 3, Figure 3). The male rats in the diabetes group did not show any statistically significant difference between the 3rd and 17th days in blood glucose levels. However, it was observed that the average blood glucose levels in the diabetes+cinnamon group decreased significantly on the 17th day (p < 0.001) (Table 4, Figure 4).

**Table 3:** The statistical evaluation of fasting blood glucose level of the female rats according to the groups.

Days	Control (mg/dL)	Sham (mg/dL)	Cinnamon (mg/dL)	Diabetes (mg/dL)	Diabetes+Cinnamon (mg/dL)
1st day	83 (80–91) aB	84 (75–90)aAB	85.5 (78–102)aA	92 (84–94)aB	87.5 (82–98)aB
3rd day	84.5 (79–87)aB	77 (73–87)bA	77 (73–82)bB	380.5 (288–427) cA	328.5 (320–397)cA
17th day	92 (88–95)aA	84 (78–91)bB	83 (76–90)bA	362.5 (280–400)cA	308.5 (158–383)cA

A, B: As a result of the Friedman test and the Wilcoxon test performed afterwards; the differences between the levels in the same column expressed with the different letters are statistically significant (p < 0.05), a, b, c: As a result of the Kruskal–Wallis H test and the Mann–Whitney U test performed afterwards; the differences between the levels in the same line expressed with the different letters are statistically significant (p < 0.05).

**Table 4:** The statistical evaluation of fasting blood glucose level of the male rats according to the groups.

Days	Control (mg/dL)	Sham (mg/dL)	Cinnamon(mg/dL)	Diabetes(mg/dL)	Diabetes+Cinnamon (mg/dL)
1st day	76.5 (67–85)ᵇᴬ	77.5 (72–80)ᵇᴬ	90.5 (75–97)aᴬ	83.5 (82–91)aᴮ	88.5 (77–97)aC
3rd day	75.5 (66–86)cA	77 (74–84)cA	76.5 (72–84)cA	373.5 (358–400)aA	338 (295–359)bA
17th day	82 (70–86)cA	80.5 (70–82)cA	79.5 (73–81)cA	359 (346–388)aA	264.5 (95–326)bB

A, B, C: As a result of the Friedman test and the Wilcoxon test performed afterwards; the differences between the levels in the same column expressed with the different letters are statistically significant (p < 0.05), a, b, c: As a result of the Kruskal–Wallis H test and the Mann–Whitney U test performed afterwards; the differences between the levels in the same line expressed with the different letters are statistically significant (p < 0.05).

#### 3. 3. Histological results

**Figure 5:**
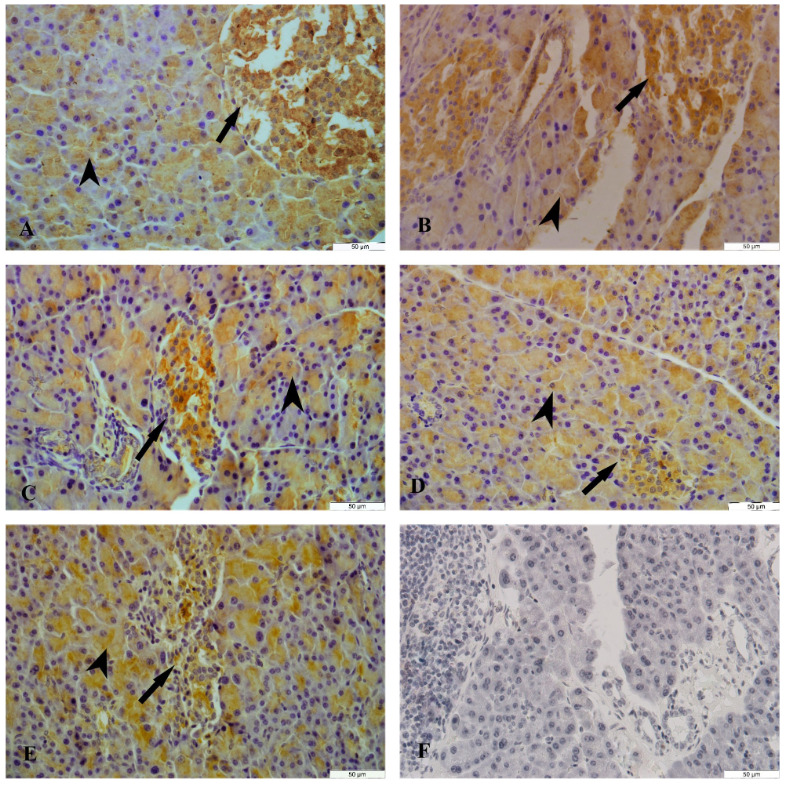
The microscopic results of female rats in all groups. A: The control group, B: The sham group, C: The cinnamon group, D: The diabetes group, E: The diabetes+cinnamon group. Arrow: The islets of Langerhans, arrowhead: The acinar, star: The pars excretoria. A, B, C, E: The triple staining, D: The Hematoxylin & Eosin staining.

**Figure 6:**
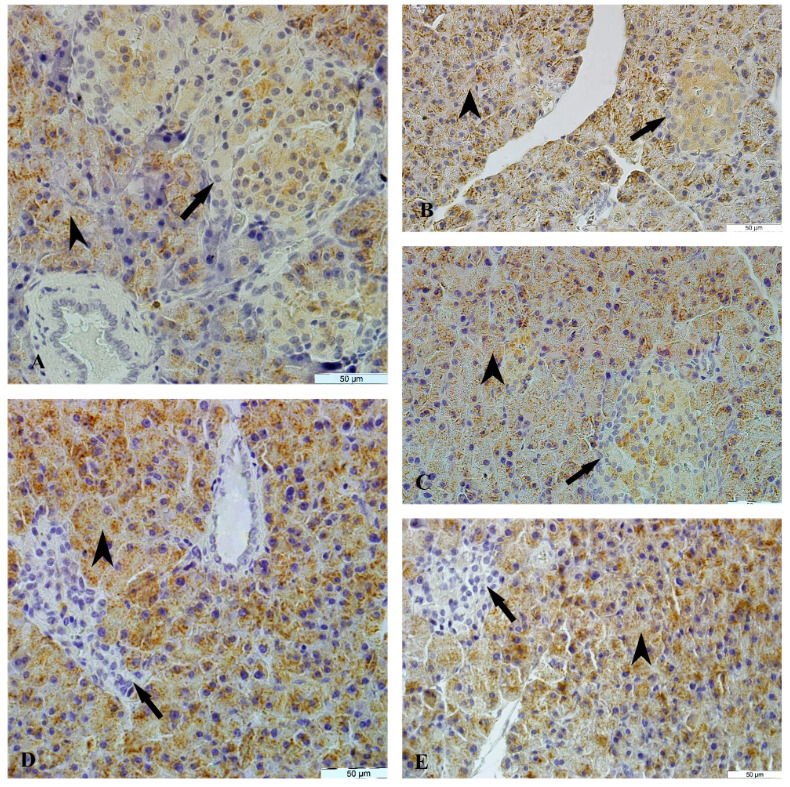
The microscopic results of male rats in all groups. A: The control group, B: The sham group, C: The cinnamon group, D: The diabetes group, E: The diabetes+cinnamon group. Arrow: The islets of Langerhans, arrowhead: The acinar, star: The pars excretoria. A, B, C, E: The triple staining, D: The Hematoxylin & Eosin staining.

The islets of Langerhans, acinar, pars initialis, pars excretoria, ductus excretorius, and blood vessels were seen at the pancreatic tissues of female and male rats. The pancreatic tissue of male and female rats was seen in normal histomorphology belonging to the control, sham and cinnamon groups (Figure 5A-C and Figures 6A-C). A significant decrease in the cell density and a particular decrease in the peripheral density were observed in the islets of Langerhans of the pancreatic tissue of female and male rats in the diabetes groups (Figure 5D, Figure 6D). The acinar of the pancreatic tissue of male and female rats in the diabetes+cinnamon groups was observed as normal and the cell density of islets of Langerhans showed a view close to normal (Figures 5E, Figure 6E). 

#### 3. 4. Immunohistochemical results

3. 4. 1. NGF Immunoreactivity

**Figure 7:**
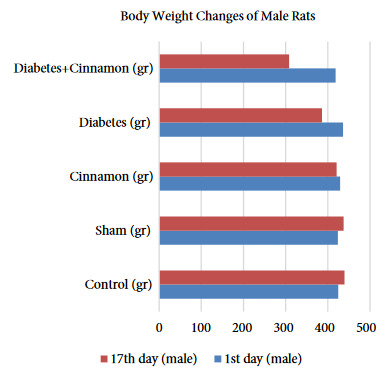
The NGF immunoreactivity in the pancreas tissue of female rats. A: The control group, B: The sham group, C: The cinnamon group, D: The diabetes group, E: The diabetes+ cinnamon group, F: The negative control. Arrow: The islets of Langerhans, arrowhead: The acinar.

**Figure 8:**
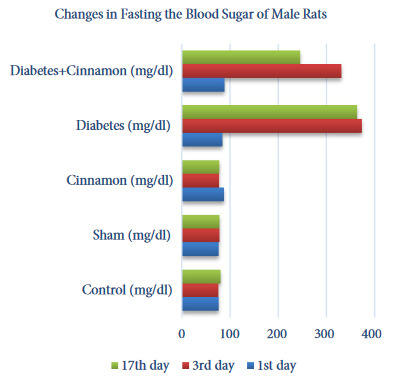
The NGF immunoreactivity in the pancreas tissue of male rats. A: The control group, B: The sham group, C: The cinnamon group, D: The diabetes group, E: The diabetes+cinnamon group. Arrow: The islets of Langerhans, arrowhead: The acinar.

The NGF immunoreactivity was determined in the endocrine and exocrine parts of the pancreatic tissue of female and male rats in all groups. A moderate diffuse cytoplasmic NGF immunoreactivity was observed in the epithelium cells of acinar, pars initialis, pars excretoria, and ductus excretorius but a strong diffuse cytoplasmic NGF immunoreactivity was detected in the islets of Langerhans on the control, sham and cinnamon groups in the female and male rats. A weak diffuse cytoplasmic NGF immunoreactivity was detected in the islets of Langerhans of diabetes group whereas a moderate diffuse cytoplasmic NGF immunoreactivity was detected in the diabetes+cinnamon group in the female (Table 5, Figure 7) and male rats (Table 6, Figure 8). The count of NGF immunoreactivity positive cells, acinar and islets of Langerhans of groups was presented in Tables 7 and 8.

**Table 5:** The semiquantitative analysis results of NGF and Trk-A immunoreactivity in the pancreatic tissue of female rats.

NGF immunoreactivity							
Area	Controln:6	Sham n:6	Cinnamon n:6		Diabetes n:6		Diabetes+ cinnamon n:6
Acini	2	2	2		2		2
Pars initialis	2	2	2		2		2
Pars excretoria	2	2	2		2		2
Ductus excretorius	2	2	2		2		2
Langerhans islets	3	3	3		1		2
Trk-A immunoreactivity							
Area	Controln:16	Sham n:16		Cinnamon n:6		Diabetes n:6	Diabetes+ cinnamon n:6
Acini	3	3		3		3	3
Langerhans islets	1	1		1		0	0

**Table 6:** The semiquantitative analysis results of NGF immunoreactivity in the pancreatic tissue of male rats.

NGF immunoreactivity					
Area	Controln:6	Sham n:6	Cinnamon n:6	Diabetes n:6	Diabetes+ cinnamon n:6
Acini	2	2	2	2	2
Pars initialis	2	2	2	2	2
Pars excretoria	2	2	2	2	2
Ductus excretorius	2	2	2	2	2
Langerhans islets	3	3	3	1	2
Trk-A immunoreactivity					
Area	Controln:16	Sham n:16	Cinnamon n:6	Diabetes n:6	Diabetes+ cinnamon n:6
Acini	3	3	3	3	3
Langerhans islets	1	1	1	0	0

**Table 7:** The comparison of count of the NGF immunoreactivity positive cells acini and Langerhans island among the groups in the female rats.

Section	Control	Sham	Cinnamon	Diabetes	Diabetes+Cinnamon
Acinar cell	1474 (-)	1475 (-)	1474 (-)	1474 (-)	1475 (-)
Langerhans islets cell	188 a	188 a	188 a	69 b	135 c
Number (unit area)	48	48	48	48	48

-; There is no statistical difference between the groups.The unit area: The total number of regions on which the slides were counted for the immunoreactivity positive cells. a,b,c; The

**Table 8:** The comparison of count of the NGF immunoreactivity positive cells acini and Langerhans island among the groups in the male rats.

Section	Control	Sham	Cinnamon	Diabetes	Diabetes+Cinnamon
Acinar cell	1177 (-)	1176 (-)	1177 (-)	1177 (-)	1176 (-)
Langerhans islets cell	248 a	248 a	249 a	49 b	128 c
Number (unit area)	48	48	48	48	48

-; There is no statistical difference between the groups.

#### 3. 4. 2. Trk-A immunoreactivity

**Figure 9:**
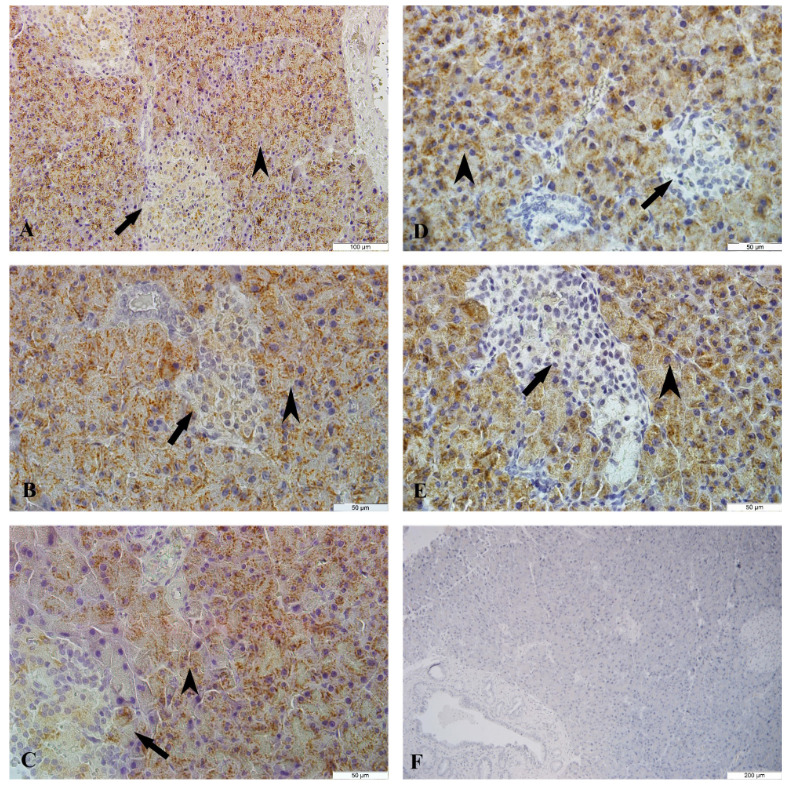
The Trk-A immunoreactivity in the pancreas tissue of female rats. A: The control group, B: The sham group, C: The cinnamon group, D: The diabetes group, E: The diabetes+cinnamon group. Arrow: The islets of Langerhans, arrowhead: The acina

**Figure 10:**
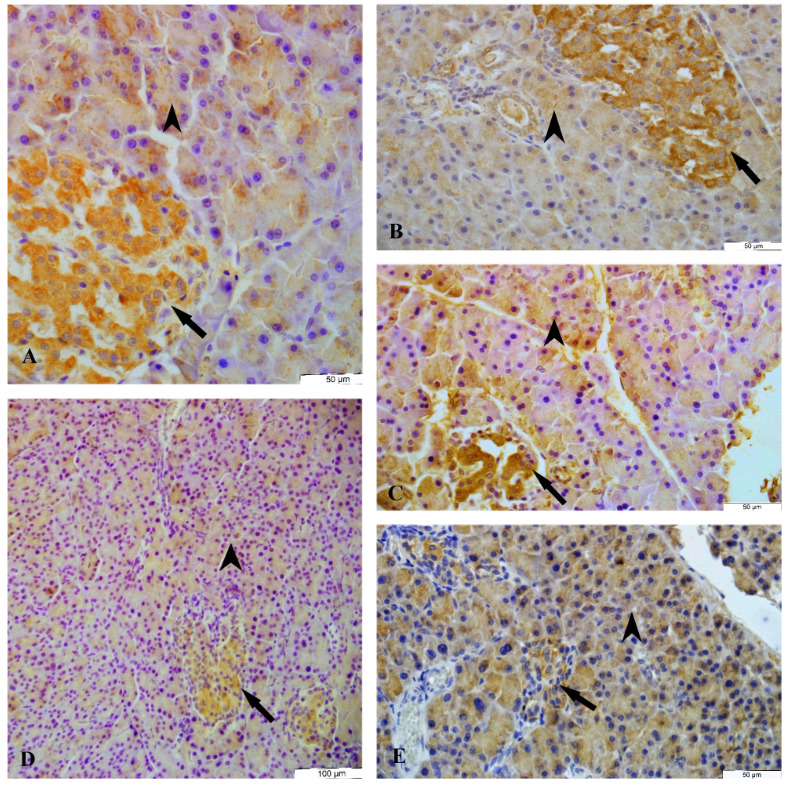
The Trk-A immunoreactivity in the pancreas tissue of male rats. A: The control group, B: The sham group, C: The cinnamon Group, D: The diabetes group, E: The diabetes+cinnamon group, F: The negative control. Arrow: The islets of Langerhans, arrowhead: The acinar.

A strong granular Trk-A immunoreactivity was determined in the cytoplasm of acinar cells and a weak diffuse cytoplasmic Trk-A immunoreactivity was detected in the islets of Langerhans of the pancreatic tissue of female and male rats in the control, sham and cinnamon groups. The Trk-A immunoreactivity in the acinar cells of pancreatic tissues and pancreatic duct in the diabetes and diabetes+cinnamon groups and the female and male rats showed similar properties with the control group. Apart from the control group, the Trk-A immunoreactivity was not detected in the islets of Langerhans in the diabetes and diabetes+cinnamon groups (Tables 5 and 6, Figures 9 and 10). The count of Trk-A immunoreactivity positive in the acinar cells and islets of Langerhans of groups was presented in Tables 9 and 10.

**Table 10:** The comparison of count of the Trk-A immunoreactivity positive cells acini and Langerhans island among the groups in the male rats.

Section	Control	Sham	Cinnamon	Diabetes	Diabetes+Cinnamon
Acinar cell	1055 (-)	1054 (-)	1054 (-)	1054 (-)	1055 (-)
Langerhans islets cell	233 a	232 a	232 a	0 b	0 b
Number (unit area)	48	48	48	48	48

-; There is no statistical difference between the groups.

**Table 9:** The comparison of count of the Trk-A immunoreactivity positive cells acini and Langerhans island among the groups in the female rats.

Section	Control	Sham	Cinnamon	Diabetes	Diabetes+Cinnamon
Acinar cell	1400 (-)	1399 (-)	1400 (-)	1399 (-)	1400 (-)
Langerhans islets cell	183 a	184 a	183 a	0 b	0 b
Number (unit area)	48	48	48	48	48

-; There is no statistical difference between the groups.

## 4. Discussion

The cinnamon was determined to regulate the adipocyt gene expression in the mouse, decrease the body weight and body lipid mass in the obese female rats, regulate the liver enzymes and lipid levels to the normal levels, increase the serum insulin levels, and decrease the glucose levels [31,32]. It was stated that the use of food additives containing the cinnamon decreased the fasting blood glucose, body mass index, body weight, and lipid mass significantly in the obese, overweight prediabetic, and type-2 diabetic male and female patients [33,34]. In our research, the female rats in the diabetes and diabetes+cinnamon groups did not show any statistically significantly difference on the 17th day of the research with regards to the live weight averages (p > 0.001). At the end of the research, both groups showed a statistically significantly decrease with regard to the first-day results. The male rats in the diabetes and diabetes+cinnamon groups showed a statistically significantly difference on the 17th day of the research with regards to the live weight averages (p < 0.001). We observed a decrease in average in the male rats, especially in the diabetes+cinnamon group. A significant level of differences in the results obtained from the male rats made us think that the application of cinnamon might have an effect on decreasing the live weight in the male rats. In addition, the cinnamon application was thought to contribute positively to the treatment and recovery process, especially by providing the weight control in the diabetic male patients. The cinnamon was pointed out to decrease the high levels of blood glucose in the female rats, regulate the lipid metabolism, repress the blood sugar by decelerating the absorption of carbohydrates from the intestines, and have a healing role by showing the insulin-like effect in the type-2 diabetes disease [35,36]. Ranasinghe et al. [37] deemed that the cinnamon had positive effects as a result of the decrease in the high blood glucose and the absence of any histochemical toxic effect in the kidney and pancreas tissues after a single dose cinnamon extract treatment in the male and female rats. They came up with the suggestion that the application dose of cinnamon was unimportant. The decrease in the high blood sugar level in the male rats in the diabetes and diabetes+cinnamon group showed similarities with some results in the literature [37]. Our results made us think that the antidiabetic effect of cinnamon might differ depending on the sex. In the histopathological evaluation of pancreatic tissues of the rats in the streptozotocin mediated diabetes, the results such as degeneration in the β cells, damage and necrosis were detected [38]. In our study no pathological results were detected in female and male rats of the cinnamon group. We thought that the cinnamon application might have roles in the cellular regeneration and tissue repair because of the decrease in the islets of Langerhans density in the male and female rats in the diabetes group and the detection in a similar level for the cell density in the control group and diabetes+cinnamon group in our study. 

The NGF was detected in the primary cell cultures to be synthesized from the β cells of the pancreatic tissue of adult and fetus. The NGF was known to arrange the sensitivity of β cells and stimulate the nerve cell development [39]. The expression of Trk-A and its ligand NGF in the pancreas during the embriyonic and fetal life suggests that the NGF and its receptor could play an important role in the development of pancreatic tissue [40]. In our research, the Trk-A immunoreactivity was determined in the cytoplasm of acinar cells and islets of Langerhans of the pancreatic tissue of female and male rats in the control, sham, and cinnamon groups. The NGF immunoreactivity was determined in the endocrine and exocrine parts of the pancreatic tissue of female and male rats in all groups. When the immunohistochemical distribution of NGF and Trk-A receptor in the pancreatic tissue was examined, it was determined that there was no difference between the sexes in terms of the severity of immunoreactivity, the regions in which it was seen. These results made us conclude that the NGF and Trk-A might have roles in providing the persistence of cell signals maintaining the survival of exocrine and endocrine pancreatic cells and they might also have roles in the synthesis and secretion of hormones in these regions. Schneider et al. [41] investigating the distribution of NGF and Trk-A in the pancreatic tissue revealed that there was a moderate diffuse cytoplasmic reaction in the exocrine pancreas, a weak diffuse cytoplasmic reaction in the nerve cords and no NGF reaction in the endocrine pancrease [41]. The Trk-A immunoreactivity in the pancreatic duct and tunica muscularis layer of arteries was stated to be weak, but the immunoreactivity in the islet cells was moderate [41]. In another research, the NGF immunoreactivity in the pancreatic tissue was notified as weak in the acinar cells and nerve cords and as moderate in the arteries and vein walls. The chronic pancreatitis patients showed increasing NGF and Trk-A immunoreactivity. The Trk-A immunoreactivity was reported to be moderate only in the nerve fibre, arterial, and venous walls. It was stated that the NGF/Trk-A pathway interactions might lead to the morphological changes and pain syndromes [42]. In our research where the diffuse cytoplasmic NGF immunoreactivity occurred at the acinus, pancreatic duct and islets of Langerhans of pancreatic tissue of the rats. We detected the Trk-A immunoreactivity in the acinus and islets of Langerhans. The granular property of Trk-A immunoreactivity in the acinar cells was similar to the literature data, but the diffuse cytoplasmic Trk-A immunoreactivity of islets of Langerhans was dissimilar to the recent literature [41]. Although the region where the Trk-A immunoreactivity was observed was the same as the literature, the type of reaction was different from the literature. This data made us think that there might be differences between the density of immunoreactivity and the regions were as they were seen. 

On the evaluation of histologic investigation of the pancreatic tissue, after the diabetes was formed, the research suggested that the decrease of cell number in the islets of Langerhans, NGF and Trk-A protein levels was as it was in the western blot analysis [43]. The live cell numbers after the NGF blockage and insulin signals in the rat pancreatic tissue were decreased compared with the control groups. Apoptosis occurred in 17% of the cells cultured in the glucose for 16 h and apoptosis was partially inhibited with the NGF and insulin [44]. Similarly, we detected a decrease in the cellularity of islets of Langerhans for the female and male rats in the diabetes group, a significant decrease in the NGF immunoreactivity and total disappearance of Trk-A immunoreactivity. These findings made us think that the streptozocin application might cause changes in the NGF and Trk-A levels by the islet cell damage and cellular damage occurring in the islets of Langerhans in the diabetes disease that negatively affected the Trk-A receptor level. In healthy individuals, the NGF was secreted and synthesized from the pancreatic β cells and regulated the Na duct density by the paracrine and autocrine pathways [45]. In the cell tissue culture study, the NGF levels were stated to increase three-fold when the β cells of female rats were treated in the glucose culture [46] and the Trk-A mRNA numbers were stated to increase 6-fold when they were applied to the NGF culture [47]. In addition, an increase in the insulin secretion depending on the increase of NGF secretion was notified [47]. In our research, we detected that the NGF immunoreactivity decreased in the islets of Langerhans of rats in the diabetes groups and increased in diabetes+cinnamon groups. The Trk-A immunoreactivity was unchanged. It might be due to the disruption of NGF and Trk-A signal pathways in the diabetes disease. It was concluded that the Trk-receptor immunoreactivity did not change in the diabetes and diabetes+cinnamon groups. Our results suggested that the cinnamon led to an increase in the NGF immunoreactivity in the islet cells and the NGF might have a role in the regulation of blood glucose in the diabetes. In addition, it was also concluded that the increase in the level of NGF could contribute positively to the prevention of diabetes-related diseases.

Based upon these data, we concluded that the cinnamon is effective in reducing the high blood glucose, significantly decrease the live weight in the diabetic male rats, change the Trk-A levels, and it also causes an increase in the NGF levels in the islets of Langerhans and it may have positive effects by increasing the NGF levels on the treatment of diabetes disease and by hindering the complications depending on the diabetes. 

**Acknowledgement and/or disclaimers, if any**

This study was supported by the Scientific and Technical Research Council of the Kafkas University (Project 2015-VF-04), Kars-TURKEY. The present study was summarized from a Ph.D Thesis and was presented at the 15th International Congress of Histochemistry and Cytochemistry (ICHC 2017), 18-21 May 2017 in Kervansaray Lara Hotel Antalya, Turkey.

## References

[ref1] İrak K Mert N Mert H Ayşin N. 2018 The effects of grape seed extract on the some enzymes and metabolites in diabetic rats Van Veterinary Journal 29 147 152

[ref2] Pipeleers D Hoorens A Marichal-Pipeleers M Van de Casteele M Bouwens L 2001 Role of pancreatic cells in the process of cell death Diabetes 50 52 57 10.2337/diabetes.50.2007.s52 11272203

[ref3] Jayaprakasha GK Rao LJM 2011 Chemistry, biogenesis, and biological activities of Cinnamomum zeylanicum Critical Reviews in Food Science and Nutrition 51 547 562 21929331 10.1080/10408391003699550

[ref4] Shreaz S Wani WA Behbehani JM Raja V Irshad M 2016 Cinnamaldehyde and its constituents, a novel class of antifungal agents Fitoterapia 12 116 131 10.1016/j.fitote.2016.05.016 27259370

[ref5] Rao PV Gan SH 2014 Cinnamon: a multifaceted medicinal plant Evidence Based Complement Alternative Medicine 10 10.1155/2014/642942 PMC4003790 24817901

[ref6] Jia Q Liu X Wu X Wang R Hu X 2009 Hypoglycemic activity of a polyphenolic oligomer-rich extract of cinnamomum parthenoxylon bark in normal and Streptozotocin induced diabetic rats Pythomedicine 16 744 750 10.1016/j.phymed.2008.12.012 19464860

[ref7] Kumar S Vasudeva N Sharma S. 2012 GC-MS analysis and screening of antidiabetic, antioxidant and hypolipidemic potential of cinnamomum tamala oil in streptozotocin induced diabetes mellitus in rats Cardiovascular Diabetology 10 11 11 10.1186/1475-2840-11-95 PMC3461457 22882757

[ref8] Reichardt FL 2006 Neurotrophin-regulated signalling pathways Philosophical transactions of the Royal Society B 361 1545 1564 10.1098/rstb.2006.1894 PMC1664664 16939974

[ref9] Bayar M Ozer B Bestas A Ceribası S Ozercan I. 2010 Effects of anti-NGF on apoptosis in rats with experimentally induced sepsis model Turkiye Klinikleri Journal of Medical Sciences 30 1127 1133

[ref10] Schäper C Gläser S Groneberg DA Kunkel G Ewert R 2009 Nerve growth factor synthesis in human vascular smooth muscle cells and its regulation by dexamethasone Regulatory Peptides 157 3 7 19596029 10.1016/j.regpep.2009.07.004

[ref11] Ieda M Fukuda K Hisaka Y Kimura K Kawaguchi H 2004 Endothelin-1 regulates cardiac sympathetic innervation in the rodent heart by controlling nerve growth factor expression The Journal of Clinical Investigation 113 876 884 15067320 10.1172/JCI19480 PMC362115

[ref12] Murase K Hattori A Kohno M 1993 Hayashi K. Stimulation of nerve growth factor synthesis/secretion in mouse astroglial cells by coenzymes Biochemistry and Molecular Biology International 30 615 621 8401318

[ref13] Johnson Jr EM Gorin PD Brandeis LD Pearson J 1980 Dorsal root ganglion neurons are destroyed by exposure in utero to maternal antibody to nerve growth factor Science 210 916 918 7192014 10.1126/science.7192014

[ref14] Trim N Morgan S Evans M Issa R Fine D 2000 Hepatic stellate cells express the low affinity nevre growth factor receptor p75 and undergo apoptosis in response to nevre growth factor stimulation The American Journal of Pathology 156 1235 1243 10751349 10.1016/S0002-9440(10)64994-2 PMC1876895

[ref15] Dang C Zhang Y Ma Q Shimahara Y. 2006 Expression of nerve growth factor receptors is correlated with progression and prognosis of human pancreatic cancer Journal of Gastroenterology and Hepatology 21 850 858 16704535 10.1111/j.1440-1746.2006.04074.x

[ref16] Gelincik A Aydın F Ozerman B Ergüven M Aydın S 2012 Enhanced nerve growth factor expression by mast cells does not differ significantly between idiopathic and allergic rhinitis Annals of Allergy, Asthma & Immunology 108 396 401 10.1016/j.anai.2012.04.006 22626591

[ref17] Artico M Bronzetti E Felici LM Alicino V Ionta B 2008 Neurotrophins and their receptors in human lingual tonsil: an immunohistochemical analysis Oncology Reports 20 1201 1206 18949422

[ref18] Levanti MB Germanà A Catania S Germanà GP Gauna-Añasco L Embryologia 2001 Neurotrophin receptor-like proteins in the bovine (Bos taurus) lymphoid organs, with special reference to thymus and spleen 30 193 198 10.1046/j.1439-0264.2001.00329.x 11534323

[ref19] Chaldakov GN Tonchev AB Aloe L. NGF 2009 and BDNF: from nerves to adipose tissue, from neurokines to metabokines. Relevance to neuropsychiatric and cardiometabolic diseases Rivista di Psichiatria 44 79 87 20066808

[ref20] Sornelli F Fiore M Chaldakov GN Aloe L 2009 Adipose tissue-derived nerve growth factor and brain-derived neurotrophic factor: results from experimental stress and diabetes General Physiology and Biophysics 28 179 183 19893098

[ref21] Davies AM Lumsden AGS Rohrer H. Neural 1987 -derived proprioceptive neurons express NGF receptors but is not supported by NGF in culture Neuroscience 20 37 46 3031542 10.1016/0306-4522(87)90004-2

[ref22] Jia Q Liu X Wu X Wang R Hu X 2009 Hypoglycemic activity of a polyphenolic oligomer-rich extract of cinnamomum parthenoxylon bark in normal and Streptozotocin induced diabetic rats Pythomedicine 16 744 750 10.1016/j.phymed.2008.12.012 19464860

[ref23] Ganda OP Rossi AA Like AA 1976 Studies on streptozotocin diabetes Diabetes 25 595 603 132382 10.2337/diab.25.7.595

[ref24] Sharafeldin K Rizvi RM 2015 Eﬀect of traditional plant medicines (cinnamomum zeylanicum and syzygium cumini) on oxidative stress and insulin resistance in streptozotocin-induced diabetic rats The Journal of Basic & Applied Zoology 72 126 134

[ref25] Luna LG McGraw-Hill 1968 Manual of histologic staining methods of the armed forces institute of pathology

[ref26] Hsu SM Raine L Fanger H 1981 Use of avidin-biotin-peroksidase complex (ABC) in immunoperoksidase techniques: a comparison between ABC and unabeled antibody (PAP) procedures Journal of Histochemistry & Cytochemistry 29 577 580 6166661 10.1177/29.4.6166661

[ref27] Shu S Ju G Fan LZ 1988 The glucose oxidase-DAB-nickel method in peroxidase histochemistry of the nervous system Neuroscience Letters 85 169 171 3374833 10.1016/0304-3940(88)90346-1

[ref28] Zhu QY 1989 Analysis of blood vessel invasion by cells of thyroid follicular carcinoma using image processing combined with immunohistochemistry Zhonghua Yi Xue Za Zhi 69 573 575 2620265

[ref29] Seidal T Balaton AJ Battifora H. Interpretation 2001 and quantification of immunostains The American Journal of Surgical Pathology 25 1204 1207 11688582 10.1097/00000478-200109000-00013

[ref30] Eliş Yıldız S Yediel Aras Ş Dağ S 2019 Immunohistochemical distribution of somatostatin in gastric tissue of diabetic rats treated with cinnamon extracts Kafkas Universitesi Veteriner Fakultesi Dergisi 25 427 433

[ref31] Cao H Graves DJ Anderson RA 2010 Cinnamon extract regulates glucose transporter and insülin-signaling gene expression in mouse adipocytes Phytomedicine 17 1027 1032 20554184 10.1016/j.phymed.2010.03.023

[ref32] Shalaby MA Saifan HY 2014 Some pharmacological effects of cinnamon and ginger herbs in obese diabetic rats Journal of Intercultural Ethnopharmacology 3 144 149 26401364 10.5455/jice.20140818050741 PMC4576807

[ref33] Vafa M Mohammadi F Shidfar F Sormaghi MS Heidari I 2012 Effects of cinnamon consumption on glycemic status, lipid profile and body composition in type 2 diabetic patients International Journal of Preventive Medicine 3 531 536 22973482 PMC3429799

[ref34] Liu Y Cotillard A Vatier C Bastard JP Fellahi S 2015 A dietary supplement containing cinnamon, chromium and carnosine decreases fasting plasma glucose and ıncreases lean massin overweightor obese pre-diabetic subjects: a randomized, placebo-controlled trial Plos One 10 10.1371/journal.pone.0138646 PMC4583280 26406981

[ref35] Kim H Hyun HS Choung YS 2006 Anti-diabetic effect of cinnamon extract on blood glucose in db/db mice Journal of Ethnopharmacology 104 119 123 16213119 10.1016/j.jep.2005.08.059

[ref36] Cheng MD Khun P Poulev A Rojo LE Lila AM 2012 In vivo and ın vitro anti diabetic effects of aqueous cinnamon extract and cinnamon polyphenol-enhanced food matrix Food Chemistry 135 2994 3002 22980902 10.1016/j.foodchem.2012.06.117 PMC3444749

[ref37] Ranasinghe P Perera S Gunatilake M Abeywardene E Gunapala N 2012 Effects of cinnamon zeylanicum on blood gucose and lipids in a diabetic and healthy rat model Pharmacognosy Research 4 73 79 22518078 10.4103/0974-8490.94719 PMC3326760

[ref38] Mohammadi J Naik PR 2012 The histopathologic effects of Morus Alba leaf extract on the pancreas of diabetic rats Turkish Journal of Biology 36 211 216

[ref39] Polak M Scharfmann R Seilheımer B Eisenbarth G Dressler D 1993 Nerve growth factor induces neuron-like differentiation of an insulin-secreting pancreatic beta cell line Proceedings of the National Academy of Sciences 90 5781 5785 10.1073/pnas.90.12.5781 PMC46806 8516328

[ref40] Miralles F Philippe P Czernichow P Scharfmann R 1998 Expression of nerve growth factor and its high-affinity receptor Trk-A in the rat pancreas during embryonic and fetal life Journal of Endocrinology 156 431 439 10.1677/joe.0.1560431 9582499

[ref41] Schneider MB Standop J Ulrich A Wittel U Friess H 2001 Expression of nerve growth factors in pancreatic neural tissue and pancreatic cancer Journal of Histochemistry & Cytochemistry 49 1205 1210 11561004 10.1177/002215540104901002

[ref42] Friess H Zhu ZW Di Mola FF Kulli C Graber HU 1999 Nerve growth factor and its high-affinity receptor in chronic pancreatitis Annals of Surgery 230 615 624 10561084 10.1097/00000658-199911000-00002 PMC1420914

[ref43] Sposato V Mannı L Chaldakov GN Aloe L 2007 Streptozotocin-induced diabetes is associated with changes in NGF levels in pancreas and brain Archives Italiennes de Biologie 145 87 97 17639781

[ref44] Navarro-Tableros V Soto MC Garcia S Hiriart M. 2004 Sa´nchez- Diabetes 53 2018 2023 15277381 10.2337/diabetes.53.8.2018

[ref45] Vidaltamayo R Sánchez-Soto MC Hiriart M. 2002 Nerve growth factor increases sodium channel expression in pancreatic b cells: implications for insulin secretion Federation of American Societies for Experimental Biology 16 891 892 10.1096/fj.01-0934fje 12039870

[ref46] Rosenbaum T Vidaltamayo R Sánchez-Soto MC Hiriart MA 1998 Pancreatic b cells synthesize and secrete nerve growth factor Proceedings of the National Academy of Sciences 95 7784 7788 10.1073/pnas.95.13.7784 PMC22756 9636228

[ref47] Rosenbaum T Sánchez-Soto MC Hiriart MA 2001 Nerve growth factor increases insulin secretion and barium current in pancreatic b-cells Diabetes 50 1755 1762 11473035 10.2337/diabetes.50.8.1755

